# Extra Supplement—The Nursing Mirror

**Published:** 1890-11-15

**Authors:** 


"The Hospital" Nursing Mirror.

Being the Extra Nursing Supplement of "The Hospital" Newspaper.

All Contributions for this Supplement should be addressed to the Editor, The Hospital, 140, Strand, London, W.C., and should have the word "Nursing" plainly written in left-hand top corner of the envelope.

### En Passant.

BRIDPORT NURSE SOCIETY.—The report just issued is satisfactory. Nurse Seamark is leaving, and Nurse Agnew, who trained in Manchester, is taking her place. The number of visits paid during the year were 1,407; expenditure, £91. The society makes a special feature of its loan department of all sorts of nursing appliances.

DUNDEE NURSES.—The annual meeting of the Dundee Sick Poor Nursing Society was held last month, Ex-Provost Moncur presiding. The society was inaugurated just a year ago, and there are three nurses, Miss Mackay, Miss Bryde, and Miss Smith. The following is a summary of the work accomplished from March 6th to September 30th:—Cases nursed, 136; visits paid, 3,440; recovered, 64; transferred to hospital, 5; died, 33; removed for other causes, 12; still on the books, 22; total, 136. The following list shows by whom the patients were sent: Medical men, 27; clergymen, 10; district visitors and biblewomen, 51; Royal Infirmary, 2; patients or their friends, 46. The report concludes as follows: The services of the nurses have been much sought after.

BELFAST NURSES.—First Scotland, then Ireland; from Dundee to Belfast is a far cry, but the news is much the same. At the quarterly meeting, the general superintendent's report stated: Our nurses are in attendance upon 141 sick men, women, and children; of this number 75 are sufferers from wounds, burns, and abscesses, requiring daily dressing and care from the nurses. The nine reports you have heard read by the superintendents of the districts have explained how very serious has been the illness in many cases, and how great an amount of alleviation we have been enabled to give. The number of patients all summer has been greater than usual, many of them being victims of the influenza epidemic. Nurse Pauley resigned her post on August 1st to go to Australia. We have engaged in her place Nurse Darroch, who has had three years' training at the union infirmary under Miss Pirrie. Her Royal Highness the Princess of Wales, president of the National Pension Fund for Nurses, having graciously promised to present the certificates of membership to the first thousand nurses who joined the National Pension Fund, added to this kindness by inviting the nurses to a garden party at Marlborough House. Fortunately, the fine summer weather caused a diminution in sickness in our city at the time, so we were able to give Nurses Spence and West their holiday, which permitted them to avail themselves of the Princess's invitation. The nurses were presented one by one to Her Royal Highness, who handed them their certificates. The Prince of Wales then addressed them, saying, in the course of his speech, that the nurses present had come from one hundred and fifty different institutions. One nurse had come all the way from Gibraltar. The Prince stated that London Hospital, Guy's, St. Mary, and King's College Hospitals, as well as others in London and the country have become affiliated to the fund, and many pay one-half of the necessary premiums to secure a pension to their nurses. The Prince remarked that the success of the National Pension Fund for Nurses was so phenomenal as to prove that women can and will attend to their own interests, and provide for old age and incapacity when the day's work is over. Our nurses greatly enjoyed their visit to Marlborough House, and to London. I must add that the arrangements made by the Committee of the National Pension Fund for the comfort and pleasure of the nurses were admirable."

"NURSES' FOOD, HOURS OF WORK, AND RELAXATION" will be the title of Mr. Burdett's paper to be read before the members of the Hospitals Association, at the Court Room of St. Thomas's Hospital, on Thursday evening, November 20th, at eight o'clock, when Sir Andrew Clark, Bart., M.D., F.R.S., will preside. A representative meeting and full discussion may be anticipated. Tickets may be obtained on written application to the Honorary Secretaries, Hospitals Association, Norfolk House, Norfolk Street, Strand, W.C.

TAUNTON VICTORIA INSTITUTE.—The Taunton and Somerset Hospital put its house in order last year, and started a nursing institution; and the first report of the new state of affairs is now out, and so far as the institution goes is highly satisfactory. The hospital accounts still show an expenditure too large for the income. The institute staff now consists of 23 nurses and 3 probationers. From various causes there have been, during the year, nine resignations. The classes for instruction of the nurses in elementary physiology and anatomy have been continued by the house surgeon, as also those in general nursing by the lady superintendent. During the year, in addition to supplying a staff of nurses to the hospital, the institute has sent out nurses to 78 private families, and their services have been highly appreciated. Mr. B. W. Housman, of Birmingham, has been appointed house surgeon, in place of Mr. Cosens, resigned.

LADIES ON COMMITTEES.—It is amusing to note the number of letters we have received objecting to ladies' committees for hospitals; all matrons seem to think that the ladies would worry around and cause extra work and give no help. Possibly this might sometimes be the case; but, on the other hand, we know several hospitals which could not get on at all without their ladies' committee, for the gentler sex it is who furnish all the nurses' rooms, provide decorations for the wards and comforts for the nurses, and extract contributions from crusty old bachelors. The model committee consists of men and women, when the work that comes before it relates to both sexes; then, when a purely feminine question comes forward, a sub-committee of women can be appointed to sit on it. But it is a great mistake for matrons, sisters, and nurses to resent the presence of ladies on hospital committees, and shows a feminine failing we would fain hope the nursing world was free from. Women can work together in harmony; and if one of their number does belong to the old-fashioned, narrow-minded, and fussy school, well, surely the women of to-day are large-minded and patient enough to put up with the follies of their erring sisters.

TO OUR CORRESPONDENTS.—It constantly happens that our readers send letters or queries to us without enclosing their name and address, and we wish to take this opportunity of stating that *no* anonymous communication is ever heeded; it may be the most innocent query, but still a rule is a rule, and we never depart from ours which states that every communication must be accompanied by the writer's full name and address as a guarantee of good faith. Another point we wish to impress on our readers is that The Hospital goes to press on Wednesdays, and that news received later than Tuesday morning cannot be inserted in that week's number. In fact we like best to hear from our correspondents on Monday, but though we do our best to insert news and answer letters promptly, we fear our correspondents have sometimes been disappointed at delay, not understanding how early in the week our printing has to be done, owing to the ever-increasing circulation of The Hospital.

### Lectures on Surgical Ward Work and Nursing.

By Alexander Miles, M.B. (Edin.), C.M., F.R.C.S.E.

### Lecture III.—ANTISEPTICS IN COMMON USE.

Corrosive Sublimate Lotion.—This is perhaps the most commonly used antiseptic in the present day. It is a preparation of mercury, and goes by various names. You should know all these names, as you may be asked to prepare this lotion by any one of them. It is called "corrosive lotion," "perchloride," "bichloride," "sublimate," and sometimes simply "mercurial lotion."

The perchloride of mercury is a white crystalline substance, and is extremely poisonous. The lotion is made by dissolving this powder in distilled water. It is antiseptic in very much diluted forms, up to 1 in 5,000 being often used. The commonest strength in use, however, is 1 in 2,000. It is customary to keep the ward stock of 1 in 1,000, and to dilute it with equal parts of tepid water when 1 in 2,000 is required.

It is unfortunate that corrosive sublimate destroys metallic instruments by corroding them. This somewhat restricts its use, and you should always bear this property in mind, as otherwise you may destroy expensive and delicate instruments by purifying them with it. On this account also, tin basins must not be used for corrosive lotion, the tin being instantly corroded, losing its polish, and turning the lotion black. You must use glass or porcelain dishes, or, better still, basins covered with a layer of enamel.

So also with syringes. Never use a brass syringe with corrosive lotion, always a glass or vulcanite one.

As far as possible, avoid putting sponges into corrosive lotion. Although it does not actually destroy them, it gives them a nasty black colour which cannot be removed.

Corrosive lotion is easily rendered non-antiseptic by adding a quantity of blood or pus to it. The albumen of the blood or pus acts on the solution, forming an albuminate of mercury, which is not antiseptic. Therefore, you should change the lotion very frequently during an operation. You can tell when this action has taken place by the thick brown deposit which falls to the bottom of the basin, resembling very much the sediment of strong beef-tea. By adding a quantity of common salt to the lotion this action may be prevented.

Caution!—Corrosive sublimate is a most deadly poison if taken by the mouth. It should, therefore, always bear a prominent "poison" label, and be placed in some position where children or delirious patients cannot reach it. Like carbolic it may also produce symptoms of poisoning by being absorbed through the skin, or from a wound. The symptoms it produces are diarrhœa, vomiting, and collapse, which may be followed by rapid death. Never then apply a large moist dressing of corrosive sublimate, and be especially careful never to put macintosh outside of such a dressing to prevent evaporation, because the contact of the lotion with the skin produces irritation, and absorption rapidly follows.

### Advantages.

1. Its cheapness.
2. Being non-volatile it remains antiseptic.
3. Its extreme efficiency.
4. Its general applicability.

### Disadvantages.

1. Its poisonous properties.
2. It corrodes instruments, tins, &c.
3. It blackens sponges.

### Uses.

1 in 500.—(1) For washing out septic cavities. (Be careful not to leave any in the cavity.)
(2) For purifying the skin.
(3) For purifying the surgeon's hands.

This strength is rather irritating for the hands, but its efficiency is undoubted.

1 in 1,000.—Same uses as 1 in 500.

This is the strength which should be kept in the stock bottle.

1 in 2,000.—(1) For irrigating at operations.
(2) ,, ,, dressings, &c.

This strength may be used for almost any purpose, being efficient, safe, clean, and readily procurable. It is made by taking a quantity of 1 in 1,000 in an enamelled basin, and adding to it an equal quantity of tepid water, thus bringing the strength down to 1 in 2,000.

1 in 5,000.—(1) In ophthalmic surgery.
(2) As a vaginal douche in gynæcology, &c.

It is prepared by adding four times as much water as 1 in 1,000.

### Preparations.

1. Corrosive sublimate lotion.
2. "Yellow wash" contains 1 part corrosive sublimate to 243 parts lime water.
3. Corrosive wool (non-officinal).
4. Wood wool (non-officinal).

Many other antiseptic materials are charged with corrosive sublimate.

(3) Boracic Acid Lotion.—This, which also goes by the name of boric lotion, has the great advantage over corrosive and carbolic lotions of being practically non-poisonous. It is a saturated solution of boracic acid crystals in water. A "saturated" solution means that the water is allowed to dissolve as much as it will of the drug. Hence it is impossible to use a lotion of boracic acid which is too strong, because the water refuses to take up more than 1 in 30, this forming the usual efficient lotion. To make it, add an ounce of the crystals to a pint of *boiling* water (because boiling water dissolves more than cold), and allow it to cool, when the excess of acid falls to the bottom again in the form of crystals. Your stock lotion bottle should always have a few crystals at the bottom, as an indication that it is sufficiently strong. In many hospitals the boracic lotion is coloured pink by adding some litmus to it. This is merely that it may be readily distinguished from other lotions by its colour, and is by no means necessary for its efficiency. Boracic acid "may prevent, but cannot eradicate sepsis."

### Advantages.

1. Its cheapness.
2. Its absolute safety.
3. It is non-irritating.
4. It is said to have sedative properties—hence the name "Homberg's Sedative Salt."

### Disadvantages.

1. It is not a sufficiently powerful germicide to warrant its use in important operations.
2. It does not counteract sepsis.

### Uses.

1. To clean and dress wounds of all sorts.
2. As an eye wash.
3. As an ear wash.
4. As a mouth wash or gargle.
5. As a nasal douche.
6. Is particularly useful for washing out the bladder.
7. As a vaginal or uterine douche.

For all these purposes it should be used tepid, and it is better to heat the full strength lotion than to add hot water, as the latter method diminishes its strength.

### Preparations.

1. Boracic lotion.
2. Boracic ointment.
3. Boracic lint.
4. Boroglyceride.
5. Impalpable boracic acid powder.
6. Impalpable boracic acid powder with chalk, an excellent dusting powder for infants and others.

So much for lotions. Next week we shall consider some of the Powders in ordinary use.

*(To be continued.)*

### A District Nurse's Case Book.

I THINK the following notes may be useful to my colleagues. I have worked in a district for seven and a-half years, and have had twenty-four scalded and burnt cases under my care. The doctors, as a rule, after the first dressing leave the case entirely to me (of course they visit daily), but I do the actual dressings, using my own tools. The committee are kind and liberal, and one London chemist gives as an annual donation £5 worth of drugs, lint, ointment, etc., so I am not stinted in any way :—

BOY, aged 11.—Boiling kettle went over both legs. Treatment, 10 days, carron oil on lint; 20 days, carbolic oil 1 to 40. Attended one month.

GIRL, aged 5.—Set fire to night dress; lower parts burnt. Carron oil. Lingered 17 days; died of shock.

BOY, aged 15.—One leg slipped into a copper of vitriol and water. Treatment, 18 days, carron oil; 20 days, carbolic oil 1 to 30. Attended 28 days.

BOY, aged 2.—Upset boiling kettle over hand. Treatment, carron oil. Attended 14 days.

BOY, aged 11.—Upset paraffin lamp over arm. Carron oil, 10 days; carbolic oil 1 to 40, 32 days; boracic ointment, 30 days. Attended two months 12 days.

WOMAN, aged 35.—Upset lamp over hand. Carbolic oil 1 to 40. Attended nine days.

BABY.—Ditto.

GIRL, aged 2.—Teapot over hand. Carron oil, 10 days; boracic ointment, 12 days. Attended 22 days.

BOY, aged 15.—Gunpowder exploded (fireworks); face burnt. Carbolic oil 1 to 30, five days; boracic ointment, seven days. Attended 12 days.

BOY, aged 4.—Boiling kettle all over. Terrible case; put on air bed. Carron oil, two months; carbolic oil 1 to 16, three months; zinc ointment, two months; boracic ointment; two months. Attended nine months.

BOY, aged 9 months.—Boiling kettle over hand. Carron oil. Attended three weeks.

WOMAN, aged 51.—Happened on Christmas Day, eleven a.m., 1888. Fainted, fell in fire (alone in the house; laundress; her daughters gone out). Back, arm, right side from under arm to knee burnt frightfully (happily only one side); put on air bed. Carron oil, three weeks; carbolic oil 1 to 40, nine weeks; zinc lotion, two weeks; zinc ointment, two weeks (did not suit it); boracic ointment and iodine lotion, 15 months; zinc powder, one month. Attended 20 months. The shock was so great, and the depth of the wounds, we thought it impossible she could rally; but kind friends supplied her with the best of nourishment (six ounces of brandy per day, reduced to four ounces, two ounces, and none, this by doctor's orders): a hot dinner daily from a lady for three months. This accident for dressings alone cost £5 19s. 9d., a charity committee kindly helping.

GIRL, aged 9.—Slipped in boiling copper; both legs scalded. Carron oil, 14 days; carbolic oil 1 to 30, one month; boracic ointment, one month. Attended two months 14 days.

BOY, aged 11.—Saucepan of soup over leg. Carron oil, 14 days; boracic ointment, 17 days. Attended one month.

BOY, aged 5.—Same accident. Same treatment. Attended 21 days.

BOY, aged 6.—Boiling kettle over feet. Carron oil, 10 days; carbolic oil 1 to 30, 15 days. Attended 25 days.

WOMAN, aged 32.—Saucepan of soup over foot. Carron oil five days; carbolic oil 1 to 20, 10 days; boracic ointment, 10 days. Attended 25 days.

MAN, aged 29.—Slipped into boiling copper; one leg. Carron oil, seven days. Attended seven days; wife too ill to see to him. Sent to hospital.

WOMAN, aged 51.—Fell on grate; burnt finger. Carron oil, two days; bread and water poultice, seven days. Attended nine days.

BOY, aged 14.—Slipped in boiling copper; both legs badly scalded. Carron oil, 10 days. House too small, sent to hospital. Attended 10 days.

GIRL, aged 13.—Saucepan of boiling soup over foot. Carron oil, six days; boracic ointment, eight days. Attended 14 days.

GIRL, aged 4.—Boiling kettle over arm. Carron oil, five days; carbolic oil 1 to 20, 10 days; boracic ointment, three weeks. Attended one month.

Cases now on hand :—

MAN, aged 33.—Scalding water jetted over face. Carron oil, nine days; boracic ointment continuing.

BOY, aged 9.—Slipped in boiling copper; both legs. Carron oil, five days; boracic ointment, continuing.

I find boracic ointment thickly spread on lint and not changed for four or five days an unfailing remedy.

### Words of Consolation.

### LOVE.

THE greatest consoler, the greatest beautifier of life is love, whether we mean God's love to us or our love to our neighbours. It makes the heart glow and burn with compassion for the sick and sorrowful, and to receive with gratitude every gentle word and kind act offered to ourselves. And though love has such wonderful power in the world, yet no one knows what it really is. Scripture tells us it comes from God, and describes it as a flame. Yes, it is a flame, steady, bright, and pure, which lights up and warms and shines from every heart it has entered upon all around. And as the nature of fire is to grow by what it feeds on, so the more light and warmth our hearts give out to others, the brighter and stronger does the flame burn in our own breasts. In the light of love we see all failings and shortcomings and impurities in ourselves and those about us, but its fire and heat cleanses and makes them all disappear. Its warmth bends the stubborn will, and melts the hard heart, and strengthens and revives all within its reach. What a desolate world this would be without love!—how silent, how dull! Prayer without it is but an empty sound going up to heaven, while that which moves and draws the heart of one man to another and to his God is a sound full of life and soul. It is as true as mysterious that man cannot be happy alone; his joy is not complete unless it is shared by another. God in His great love made us in the likeness of Himself. For His pleasure we *are* and were created, says St. Paul, and though we have fallen away from our first purity and goodness, and, alas! frequently hate to do what He wishes to have from us, yet His burning love never fails. Many waters of coldness and ingratitude cannot quench God's love. He goes on pouring it out upon us in mercies untold. He gives us the sun and moon to cheer us by day and by night. The rain moistens the earth, and brings forth food for cattle and green herbs for the use of man, and bread which strengtheneth man's heart. All creatures wait upon God, and He giveth them their meat in due season. "That thou givest them," says the Psalmist, "they gather, Thou openest thine hand, they are filled with good. Thou hidest Thy face, they are troubled; Thou takest away their breath, they die, and return to their dust." His love and care for us is untiring as only eternal love can be. A mother's love is the most perfect picture of earthly affection, but shall not a woman forget the fruit of her womb? Yet will *I* never forget, saith the Lord.

Can anything be more cheering than to know we belong to such a loving Father, who will not let even a sparrow fall to the ground without His knowledge. Are ye not much better than they? Try then to return this great love in some measure, because if we can catch only a few sparks of His Divine fire, we shall see that He does *all things* well, and our souls will bask in His presence and glow with the warmth which He sends into our hearts. We shall be able to say, "Then first I lived when I began to love." Yes, to live the true life, the life of love, and gratitude, and joy which springs from our perfect trust in God. This life we will devote to His service, for in it is true freedom and the only happiness. Our loving King and Father not only makes our happiness in this world, but secures it in the world to come. He has prepared for us a rest and home in the Paradise of God, where there are many mansions waiting for those who love Him. Love then the Blessed Saviour whose merits have won back our first state as sons of God. Love him for all He has done for mankind in general, and for us poor wandering sad ones in particular. He will bring us out of the sorrow and darkness of this world into the glorious light, warmth, and rapture of love in the life everlasting.

### Nurses on Their Travels.

### JOHANNESBURG AGAIN.

September, 1890.

The appearance of my scribble-scrabble in The Hospital has created a storm in a teacup here. The *Diggers' News* is very angry with the *Pall Mall Budget* for reproducing what it calls my crude opinions about the nursing of the Johannesburg Hospital. But I didn't give any opinion ; I only related what I saw, leaving people to draw their own conclusions therefrom. Some amusing letters on this subject have appeared in the *Digger*. One correspondent says that my indiscreet betrayal of local weaknesses will injure the position of the country at home and abroad ! You may imagine how important I felt after reading this, and realising that the fate of nations trembled at the end of my pen ! Another writer to the *Digger* declares that the nuns have spent nine years on the battle-fields of Europe. If this be true, they seem to have lost a great deal of time, and six months at the London would have done more for them. This home is loaded with debt, and has only contrived to exist during the past six months, because the leading doctors, four of whom are on the hospital visiting staff, send their more serious cases, especially their operation cases, to the home instead of to the hospital. The fact speaks for itself. No one who has spent a few months in this magnificent climate can help believing that the epidemics which have raged here have been the result of accident. Overcrowding, the absence of all sanitary arrangements, and the reckless, feverish life people led during the boom, are quite enough to account for double the amount of sickness. The nurses' homes exist only because they supply a want created by the bad management of the hospital. They would disappear in a few months if that institution were efficiently nursed, and sent nurses out to private cases. It will seem incredible to English people that a body of women who undertake to nurse a large hospital should refuse to be present at most operations; and that after an abdominal section, the patient must either be made ready for bed by the doctors, or left to unskilled male attendants. But the question is wholly a sectarian one. One of the hospital doctors told me that if he or any other member of the visiting staff were to complain that his patients were badly nursed, his complaint would most probably be treated as a Protestant attack, and he would be asked to resign. The nuns are such good women, and mean so well, that it is a thousand pities that there should not be a compromise, for whilst keeping the general direction of the hospital, they would have trained nurses in the ward who would attend operations and wash patients without being haunted by the spectre of possible impropriety. Next week, four of the five English nurses who left England will start for Kimberley—Miss Hickman, Miss Welby, Miss Sleeman, and I, having been fortunate enough to obtain work under Sister Henrietta. The house in which we live is let, and Miss Mollett, with the six remaining nurses, are to squeeze into a house containing four bedrooms, one sitting-room, a tiny kitchen, and a microscopic pantry. The two wards which have been built during the last eight months are detached, and are fairly comfortable, being nicely fitted up, clean, and cool. From a nurse's point of view, these wards may be considered as miracles of inconvenience—everything that has to be emptied must be carried across a rough, often muddy, yard—and food must be carried across from the tiny kitchen which supplies both the wards and the nurses; here, too, everything is washed up. But the patients are well looked after by Miss Kirkpatrick, and as little as possible of the discomfort arising from the inconceivable muddle and confusion of the domestic arrangements falls on the sick. But undesirable as the home is in more ways than one, it is far preferable to the hospital, and should the editor of the *Diggers' News* fall ill, and need poultices, I strongly advise him to apply to Miss Kirkpatrick, rather than to the nuns. Rose Blennerhasset.

*(To be continued.)*

### Everybody's Opinion.

*[Correspondence on all subjects is invited, but we cannot in any way be responsible for the opinions expressed by our correspondents. No communications can be entertained if the name and address of the correspondent is not given, or unless one side of the paper only be written on.]*

### COTTAGE NURSES.

I don't want "cottage nurses" to be spoilt by receiving too much public notice, but at the same time in justice to Miss Broadwood, I must take a share of the blame of supplying them. I do not know what Plaistow would do without them now, they are in such request. "Go to the Nurses' Home and ask for a village nurse, and then you'll be sure of a good one," was a doctor's instruction to his patient this week. Another doctor writes to me in reference to a typhoid case with acute delirium : "I must add the expression of my gratitude for the help St. Mary's Nurses' Home has been to me. But for your nurses the boy must have gone. Nurse ——, by her unremitting care and attention, has brought the boy safely round." Yet another writes in reference to nursing arrangements : "Thank you for the services of your nurses to my patients which I much appreciate." Plaistow is poor, and no one gives us trained nurses, so we nurse with these "cottage nurses" only, under supervision. Theoretically, it may be a bad thing to do ; practically, it answers capitally. You would only have my word for it if I quoted what the patients say of their nurses. The time of learning is regretably short, but good use is made of it, and the nurses receive a great deal more individual attention than they would do in hospitals. We can't all afford highly-trained butlers and footmen, and parlour-maids. As well forbid us to take instead a respectable general servant, as to deride villages for taking "cottage nurses" when they can't afford hospital trained nurses. Perhaps your readers might tell us in how many villages highly-trained hospital nurses have failed to succeed. Even one of my village nurses said to me to-day : "Oh, Sister, it will be dull when I go back to my village ; there's hardly anyone to nurse there."

Katherine Twining.

St. Mary's Nurses' Home, Plaistow, E.

### MALE NURSES.

"One of These" writes: The letters in the "Nursing Mirror," both from a Male Nurse and the Secretary of the Hamilton Association, are interesting to many male nurses besides myself, I have no doubt, giving, as it does, to those outside London some slight idea of the association and its working. I should very much like to see a copy of its rules, if that were possible, and cannot help thinking there must be some very great advantage to male nurses that may work from some such association. Unfortunately for any male person that has taken up nursing otherwise than in London, there is nothing of the kind that I know of, and we are left to the tender mercies of the private nursing institute proprietor, or have to work as best we may on our own account. I don't know what the experience of others may be, but I think I may safely say from my own that the Hamilton Association must be bad indeed if it comes in any way near some of those where the proprietors are simply working for the profits or earnings, without providing either the semblance of training, suitable accommodation for those they employ, or anything like satisfactory food, and offer in wage a sum one would be ashamed to name; and I for one should certainly like to see some such association in every city or large town at least. I trust ere long that the whole question of the proper training of males as nurses for the many cases where they are absolutely necessary will occupy a prominent place. It rightly should in the attention of those that have the best interests of the nursing question at heart; and that Hamilton Associations, or something of the kind, will also have a place in the question, with the humble though firm belief gained by experience, that thereby it will be adding much to nursing as a whole, by perfecting one of its branches.

### Death in Our Ranks.

ON November 6th, at the Fever Hospital, Birkenhead, Nurse M'Coy died of typhus fever, after a protracted period of suffering. Nurse M'Coy was attached to the Nurses' Home, Grangemount, and the Lady Superintendent there speaks of her as "a noble-hearted woman, amiable, gentle, and loving." Nurse M'Coy was the first to volunteer for service in the wretched hospital—which has now become known as the Birkenhead scandal—when the typhus outbreak occurred. Both Nurse M'Coy and her fellow worker contracted the diseases; and no wonder, for the place was in a most unsanitary condition, and the nurses had to do all the heavy work. The death of Nurse M'Coy adds another name to the long list of modern martyrs.

### Appointments.

ELGIN HOSPITAL.—Nurse J. Paterson, of the Edinburgh Royal Infirmary, has been appointed matron of the Elgin Hospital.

LONDON HOSPITAL.—Miss Anna Tillyard has been appointed Sister of the George wards, and Miss Agnes Watt, Sister of the Sophia wards; both trained at the London.

NAPLES HOSPITAL.—Nurse Roy, who has lately finished her training at the Royal Infirmary, Edinburgh, is going to the Naples Hospital, where she will be the only English-speaking nurse. The matron of this hospital is also English.

INDIAN NURSING SERVICE.—The following appointments have been made: As Lady Superintendent, Miss J. M. C. Barker; as Nursing Sisters, Miss A. S. Bates, Miss E. Bennett, Miss A. Bolster, Miss M. Carlisle, Miss L. M. Derham, Miss E. Fawcus, Miss M. Hislop, Miss J. M. Hughes, Miss E. L. Jardine, Miss C. M. Johnstone, Miss E. C. Kayes, Miss D. M. Moore, Miss A. M. Murray, Miss M. Pearse, Miss R. C. Skinner, Miss I. E. Stanistreet, Miss F. G. Stoney, and Miss C. L. Still, all trained in England, and now going to India; and Miss J. M. James and Miss E. Kelly, who are in India, and have received their appointments direct. This makes an addition of twenty-one to the Indian Nursing Service, showing how greatly the services of these women have been appreciated.

ERRATA.—Miss Burgess writes that she was trained at St. Thomas's, and only acted as "Sister" under Miss Gibson. It was Mrs. (not Miss) Thomas who took the Radford medal.

### Examination Questions.

OUT of the sixty-four answers received to last month's question, not one was incorrect, though a few were more or less faulty. For instance, the nurse who stated that the temperature of a typhoid ward should be from 65 to 70 deg., was evidently not aware of the modern cold treatment for fevers. Another nurse who sent us a dissertation on "Points on Nursing All Infectious Fever Cases" had better have stuck to the one point of the question which had no reference whatever to other fevers than typhoid. The best answers came from Marylebone Infirmary, North Malvern, Nurse Edith, Bury St. Edmunds, Louth, Nottingham, London Hospital, Derby, Bradford, and Manchester. All these were worthy of prizes, but as we had only one book ("The Theory and Practice of Nursing," by Percy Lewis), at our command, it was finally decided to send that to Nurse Gough, of the Liverpool Institute, whose answer will be printed next week. The question for November is:—"How would you administer to a patient (1) castor oil, (2) cod-liver oil, (3) effervescent medicine, (4) a powder, (5) a pill." Answers must be short and clear, written on one side of the paper only, and must reach our office by December 6th. Each answer must be accompanied by the writer's full name and address, and the word "Nursing" must be written on the envelope.

### Notes and Queries.

TO CORRESPONDENTS.—1. Questions may be written on post-cards. 2. Advertisements in disguise are inadmissible. 3. In answering a query please quote the number. 4. If a private answer is desired, a stamped addressed envelope must be forwarded.

NOTICE.—We must really ask our correspondents to be more particular in enclosing name and address. Constantly we receive queries or letters with no name attached.

### Queries.

(7) *Massage.*—A. G. K. is desirous of taking a course of lessons in massage and battery, and would be greatly obliged if the editor of THE HOSPITAL will recommend her to the best place for training; what are the terms or fees, and how long would it take to become proficient in the art?

(8) *Diet.*—K. G. A. is now nursing a lady who is suffering from a severe form of dyspepsia, and her diet is restricted to milk, farinaceous foods, and vegetables. Could any of your readers tell me how to vary this? I should like some receipts for white soups, or a book on sick cookery.

(9) *Tetanus.*—Will any nurse who has nursed a case of tetanus that recovered give a history of the case?—*J. G.*

(10) *Insurance.*—Ought a nurse to insure her own effects (such as receipts, stock, &c.) against fire, or would the hospital make good any loss she suffered in this way?—*Scot.*

(11) *Amusement.*—How can I amuse male scarlet-fever convalescents? —*Fever Nurse.*

### Answers.

*Sister.*—Next week.

*A. E. G.*—The Kyrle Society has a musical branch which holds concerts in hospitals, infirmaries, workhouses, &c. Write to Miss L. James, 14, Nottingham Place, W., for further particulars.

*Nurse M.*—Many thanks for your kind letter. We know all about the infirmary in which you are working, and the many trials. But please struggle on and do your best, for the guardians really are alive to the faults of the institution, and there is a good time coming.

*Miss Murphy.*—The institution is Roman Catholic. Particulars have been sent you by post. Did you see the account of a similar institution in THE HOSPITAL for September 27th, under the heading "A Catholic Nurse's Holiday."

(7) *Massage.*—Terms and time depend on how thoroughly you are going to learn massage; one doctor asserts that two years in Germany at a cost of £100 is the only real way to learn. Apply to the Matron, National Hospital, Queen's Square, W.C., or to Mrs. Creighton Hale, 46, Madox Street, W. Perhaps our readers can give further particulars and addresses.

*Money Order, 3s.*—(F. E., Selby). must send full name, as letters have been returned through Dead Letter Office. Please address, The Publisher, 140, Strand, W.C.

(6) *Nurses' Cloaks.*—Can be had from Messrs. Wallis and Co., High Holborn, London, E.C. Thick cloth, 19s. 6d,; cheaper cloth, 12s. 6d.—*J. G.*

### Vacancies.

THE following vacancies are announced:

**Matrons.**

Cancer Hospital, Brompton (Assist.) H.M. Hospital, Stepney (Assist.)

**Superintendent.**

St. Patrick's Home, Dublin.

**Sister.**

Royal Albert Hospital, Devonport; £25.

**Nurses.**

Alexandra Hospital, Brighton; £25.

Bath Mineral Water Hospital.

Burton-on-Trent Institution; £25.

Bournemouth Institute; £25.

Braintree Union: £22.

Brighton Fever Hospital (night); £24.

Barnstaple Home; £25.

City and County Institution, Worcester.

Chester Diocesan Deaconess Institution; £24

Children's Hospital, Highgate; £25.

Convalescing Fever Hospital, Darenth (temp); £22.

Dewsbury Infirmary; £24.

Dorset County Hospital, (night); £22.

Eastern Fever Hospital, Homerton; £30.

Easter Ross Combination, Tain; £30.

Eye and Ear Infirmary, Liverpool; £22.

General Nursing Institute, Covent Garden.

Huddersfield Home.

Jersey Institute; £25.

Jessop Hospital, Sheffield (night); £25.

Kensington Infirmary; £20.

Leicester Institution; £25.

Middlesboro' Fever Hospital.

Middlesboro' Association.

Manchester and Salford Institution; £25.

Newport (I.W.) Institute; £20.

Paddington Infirmary (night); £20.

P.M.V. Homes, Addlestone £15.;

Queen's Institute, Cardiff.

Rural Nursing Association.

Southport Infirmary; £26.

St. Mark's Hospital, E.C.; £30.

St. Leonard, Shoreditch; £20. Temp., £22; Assist., £17.

Salop Infirmary; £25.

Stephen Monckton Home, Kent.

Suffolk General Hospital (night) £20.

Tonbridge Union; £25.

Victoria Home, Bournemouth; £25.

Weston-super-Mare Hospital; £25.

Wilson Institution, W.; £30.

Worthing Institute; £25.

**District Nurse.**

Hooper's Agency, Upper Baker Street, W.

**Probationers.**

Chest Hospital, City Road, E.C.

Eye and Ear Infirmary, Liverpool.

Grantham Hospital.

Middlesboro' Association.

Samaritan Hospital for Women, Nottingham.

St. Leonard, Shoreditch; £10.

Wallasey Cottage Hospital.

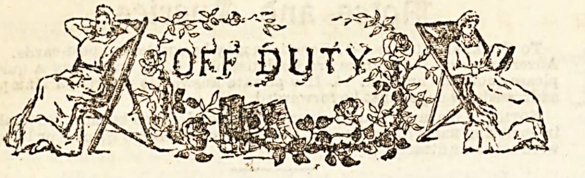

### Theatricals.

With the approach of Christmas comes the appearance of those mysterious little pamphlets with dull yellow covers which clerks con on the top of 'busses, and even nurses study while padding splints. "Please hear me my part" is the frequent demand on all sides, and conversation is flavoured with references to scenes, costumes, and get-ups. The cheerful patient is on the high road to recovery, hence the popularity of theatricals, concerts, and charades in the hospital world. Student or nurse turns in at French's, 89, Strand, and selects a short farce or book of charades, or country folks can write for the useful "Guide to Selecting Plays," price 1s. 0d., which French now publishes. This guide tells you exactly how many acts, what time the representation takes, how many characters, and what scenery is necessary. With its help it is perfectly easy to choose a play which will amuse the patients and meet the requirements of the amateur company. Some of these favourite plays to be obtained in French's Acting Edition (6d. each) are "Case for Eviction," "Domestic Economy," "Coming Woman," and "Christmas Boxes." This last, besides being seasonable, is short and easy of representation. There are five characters, two male and three female; the costume is modern morning dress, and the scene a drawing-room. It is delightful to see a nurse's face of dismay when she is first asked to help in a play; the notion of added work is too much for her. But soon she enters into the spirit of the thing, and looks forward to the rehearsal. The change of thought is as good for her as the representation is for the patients. One word of advice to amateur actors in hospitals: let your play be very short and easy to produce, and let it be amusing. Don't grudge the hiring of wigs or costumes when necessary; the actor who is well made up has already half secured a triumph.

Where theatricals are rather beyond the scope of the entertainers, charades are very useful. You needn't be so particular about scenery for charades, and there is room for invention on the part of all the actors. French publishes two books of excellent charades, "Comic Charades," price 1s., and "Charades for Acting," price 1s. But there is no absolute necessity if your entertainment is an almost impromptu one to learn charades off by heart; the chief point is to choose a good word, and be sure that your company enter into the spirit of the thing, and are not nervous or self-conscious. "Hottentot" is a good word, and the second syllable can be given by the singing or recitation of the "Ten little niggers," which makes an agreeable diversion. Masks cut out of crape can be used to avoid the blacking of faces, or 5th of November masks can be bought for 1d. each. "Bandage" is a suitable word for ward charades, and "plaintiff," "speculate," and "Friday" offer opportunities for fun and frolic. Patients will be found very apt in guessing if simple words are chosen, and they will enjoy the slight effort demanded of them, more than if they were merely spectators of a play. A very common mistake in hospitals is to have too grand and too long a performance done by outsiders. The doctors, students, and nurses, with a few of the friends of the hospital, or members of the committee, ought to take the chief parts, for there is double interest to the audience when they know the actors.

### Lectures.

The Brown "Animal Sanatory Institution."—A course of five lectures, free to the public, as required by the will of Mr. Brown, will be delivered by the Professor-Superintendent (Victor Horsley, Esq., M.B., F.R.S.), in the Theatre of the University of London, Burlington Gardens, W., on the afternoons of November 14th, 21st, and 28th, and of December 5th and 12th, commencing each day at five o'clock. Subject: "The Arrangement and Function of the General Nervous System in Relation to Epilepsy." These lectures ought to be of the greatest interest to mental nurses and asylum attendants.

### "A Voice from the Children."

In a ward in a children's hospital,
In this city of ours somewhere,
On a small white bed,
And all but dead,
They tenderly laid her there.

Only a child run over;
A little waif and stray,
Such as one may meet
In a London street
In plenty, every day.

Only a child run over;
So echoed the gathering throng;
And one pitying look,
Her ladyship took,
Then her horses and carriage dashed on.

Not lifeless, but still unconscious.
And the skilful surgeon said:
"I greatly fear
The injury's here,"
And he gently touched her head.

Unconscious, still unconscious,
Through the hours of night she lay,
And a young nurse kept,
While the gay world slept,
Close watch, till the dawn of day.

Suddenly a stained glass window
Was flooded with sunshine bright,
And a Christ child fair
That was painted there,
Shone with a wondrous light.

Just then, the blue eyes opened,
Gazing round with a vacant stare,
Then a peaceful smile
The face bore awhile
As the eyes caught the picture there.

And then the pale lips murmured,
And the nurse to her side drew near,
And knelt by the bed,
As the young child said:
"Lady, does God live here?"

"God is in heaven, darling,

And this a sick ward, you know,"

The child shook her head,

As slowly she said:

"Then this must be heaven below."

All day the small sufferer linger'd,
To be tended with hands of love,
Till the soul took flight
In the early night,
To its God in the heavens above.

Oh, women that serve the children,
The nurselings of want and woe,
How nobly true
Is the work you do,
When you help to make heaven below.

A. G. C.

### Amusements and Relaxation.

**N.B.—THIRD QUARTERLY WORD COMPETITION**

**Commenced Oct. 4, 1890; ends Dec. 27, 1890.**

Three prizes of 15s., 10s., 5s., will be given for the largest number of words derived from the words set for dissection.

Proper names, abbreviations, foreign words, words of less than four letters, and repetitions are barred; plurals, and past and present participles of verbs, are allowed. Nuttall's *Standard* dictionary only to be used.

N.B.—Word dissections must be sent in WEEKLY not later than the first post on Thursday to the *Prize Editor*, 140, Strand, W.C., arranged alphabetically, with correct total affixed.

The word for dissection for this. the SEVENTH week of the quarter, being "ARMCHAIRS."

| Names. | Nov. 6th. | Totals. | Names. | Nov. 6th. | Totals. |
|---|---|---|---|---|---|
| Jenny Wren | 14 | 201 | Liz | 10 | 136 |
| Tinie | — | 55 | Checkmate | — | 76 |
| Agamemnon | 12 | 200 | Silver King | — | 163 |
| Patience | 13 | 200 | S. Anthony | — | 76 |
| Ecila | 13 | 200 | Quackah | — | 75 |
| Lightowlers | 13 | 193 | Reynard | 15 | 164 |
| Rouge | — | 89 | Sally | — | 27 |
| Wyamaris | 12 | 184 | Success | — | 61 |
| Qu'appelle | 13 | 179 | Caledonia | — | 52 |
| Nosam | 13 | 180 | Nurse Emma | 11 | 105 |
| Nurse Hilda | — | 44 | Hazel | — | 20 |
| Lady Betty | 12 | 183 | Pallas | — | 48 |
| Grenelle | — | 43 | Puss | — | 15 |
| Daisy | 14 | 169 | Shakespear | 13 | 128 |
| H. A. S. | 15 | 157 | Melita | 15 | 185 |
| A. B. C. | — | 66 | Nora | 10 | 10 |

### Notice to Correspondents.

N.B.—Each paper must be signed by the author with his or her real name and address. A *nom de plume* may be added if the writer does not desire to be referred to by us by his real name. In the case of all prize-winners, however, the real name and address will be published.

Competitors can enter for *all* quarterly competitions, but no competitor may take more than *one first prize* during the year.

